# Knowledge, Attitudes, and Practices Regarding Antibiotic Use and Resistance: A Cross-Sectional Study among Students in Israel

**DOI:** 10.3390/antibiotics12061028

**Published:** 2023-06-08

**Authors:** Keren Dopelt, Almog Amar, Nickol Yonatan, Nadav Davidovitch

**Affiliations:** 1 School of Public Health, Faculty of Health Sciences, Ben Gurion University of the Negev, Beersheva 84105, Israel; nadavd@bgu.ac.il; 2 Department of Public Health, Ashkelon Academic College, Ashkelon 78211, Israel; almogamar@edu.aac.ac.il (A.A.); nickolyonatan@edu.aac.ac.il (N.Y.)

**Keywords:** antibiotics, antimicrobial resistance, KAP, students, Israel

## Abstract

Antibiotic resistance is one of the biggest threats to human health, food security, and development. This study aimed to examine the level of knowledge and awareness regarding antibiotic resistance while comparing students from health sciences to students in other disciplines. A cross-sectional study was conducted based on the “antibiotic resistance” questionnaire developed by the World Health Organization. A total of 371 students participated in the study. All respondents had taken antibiotics in the past. A tenth had taken them on their own without a prescription, and 14% had not received an explanation regarding the use of antibiotics. The average for the knowledge questions was 15.49 ± 5.35 (out of 27). Many students mistakenly associated antibiotics with viral diseases. Despite these misconceptions, there was a high level of awareness and understanding regarding the ways to treat antibiotic resistance. Still, the awareness of the severity of antibiotic resistance was not high. Differences were found between the disciplines in general knowledge and the level of awareness and understanding about the ways to treat antibiotic resistance, where health science students had the highest scores, followed by social science students and finally, computer and management students. No differences were found in the perception of the severity of the phenomenon. This information is essential to developing educational interventions to improve knowledge, attitudes, and practices regarding antibiotic use among students, especially those unrelated to the health sciences.

## 1. Introduction 

Antibiotics have been one of the most significant medical breakthroughs in modern medicine [1], enabling the treatment of complex medical conditions and saving millions of lives to date [2]. Antibiotic resistance is one of the most prominent threats to health systems and food security in the world [3], posing a major risk to human life and public health [4]. An estimated 700,000 people die every year of infections caused by antibiotic-resistant bacteria [5,6], and the World Health Organization (WHO) predicts that this number could rise to 10 million by 2050 if new and better treatments are not found [4,7].

Antibiotic resistance has been reported throughout the world [8]. A contributing factor to its development is antibiotic overuse, leading to antimicrobial resistance [9,10]. As such resistance becomes more common among bacteria, treating bacterial diseases becomes increasingly difficult. At present, some bacteria are already resistant to almost all antibiotics [11]. As these bacteria multiply and more bacteria acquire similar resistance, the probability of contracting a disease that cannot be treated by antibiotics increases [11]. Infections caused by resistant bacteria are typically characterized by a more prolonged duration of illness and more aggressive treatment measures, with corresponding complications and mortality rates that tend to be higher than for infections caused by non-resistant bacteria. Another aspect of the harm associated with these antibiotic-resistant pathogens is the resources consumed when treating affected patients, including the loss of working days and decreased occupational productivity due to prolonged hospitalizations, all of which divert more resources to patient care and contribute to significantly higher treatment costs [12]. According to the Israeli Ministry of Health, more than 4100 patients were hospitalized due to infections caused by antibiotic-resistant bacteria in 2015 alone, with an average hospitalization duration of two weeks. The total number of hospitalization days during 2015 for these patients exceeded 64,000, and the total cost was more than 128 million NIS [13]. In addition, in this global era, antibiotic-resistant bacteria can rapidly transfer from continent to continent, thereby leading to the global dissemination of these resistant bacteria [14].

Studies have identified several public misconceptions regarding antibiotic use and resistance [15]. Many individuals are unaware of the connection between antibiotic-resistant bacteria and the use of antibiotics [16]. For example, some people who are well informed regarding when antibiotics should be used still report feeling confident about self-medicating with antibiotics left over from a prior prescription and without consulting a physician [17]. A systematic review of 54 studies focusing on the public’s knowledge and beliefs about antibiotic resistance found that some participants had heard of it [18], but most believed it referred to changes in the human body. Participants believed they were at a low risk of antibiotic resistance and argued that strategies to minimize resistance should be aimed primarily at physicians. The researchers concluded that the public lacks a complete understanding of antibiotic resistance and harbors misconceptions regarding such resistance and its causes while believing that they do not contribute to the development of this phenomenon.

Analyses of students have reported improper antibiotic use and a lack of knowledge about antibacterial substances [19]. A survey of 1200 undergraduate students in the United Arab Emirates found that 38% of the students had self-administered antibiotics without consulting with a physician in the six months preceding the study [20]. More than half of these students had taken antibiotics for a cold or sore throat. The researchers assumed that the sick students thought the antibiotics would expedite their recovery and enable them to return to their studies more quickly. In a separate study of 733 students in Ecuador [21], differences in attitudes about antibiotic use between disciplines were identified, with students from the basic sciences receiving a higher knowledge score than their peers from the social sciences. Most interviewees were knowledgeable about the use of antibiotics but mistakenly associated the patient as the source of antibiotic resistance and not the bacteria. Similarly, a study that examined knowledge and attitudes among 750 Lebanese students found that approximately 78% of respondents from health-related majors exhibited a high level of knowledge compared to only 41% of students from non-health-related majors [22].

Although the issue of antimicrobial resistance is not new, it has long been assumed that this problem could be solved by the continuous development of novel drugs. However, antibiotic development has declined alarmingly over time [23]. Returning to the “pre-antibiotic era” will render many routine infections untreatable and will seriously affect surgery, intensive care, organ transplantation, and cancer treatment, resulting in a significant increase in morbidity and mortality. Assessing the public awareness of the phenomenon is vital for efforts aimed at addressing the spread of antibiotic resistance.

This study seeks to examine the levels of knowledge, awareness, attitudes, and behaviors of students in Israel related to antibiotic resistance and the relationships among these variables while also comparing the knowledge and awareness of students in the health sciences to those of students from other disciplines. These research findings can assist policymakers in public health and infectious diseases in formulating an outreach program to improve the public knowledge and understanding of antibiotic resistance and how people can influence and mitigate this threat to global public health. No similar studies have been conducted to date in any academic institutions in Israel.

## 2. Results

### 2.1. Participants’ Characteristics

In total, 371 individuals participated in the study, of whom 74% were women, 57% were in relationships, and 27% had children. Most participants were Jewish (92%) and Israeli-born (86%). The mean age of the respondents was 28 ± 8.58 years. The differences between disciplines were analyzed using χ^2^ tests and one-way ANOVAs. Participant characteristics are summarized in Table 1.

### 2.2. Antibiotic Usage

Differences between disciplines regarding antibiotic usage were analyzed using χ^2^ tests. The pattern of antibiotic usage among study participants is presented in Table 2. All participants had taken antibiotics, with half having done so in the past year. Of these respondents, 14% had not received an explanation of how to take antibiotics from a doctor, nurse, or pharmacist, and 10% had taken antibiotics on their own using medication they already had at home. While the differences among these groups were not significant, self-medicating with antibiotics was more common among health science students.

### 2.3. Levels of Knowledge and Attitudes

Table 3 presents the levels of knowledge and attitudes toward antibiotic resistance among the study participants. The knowledge about the use of antibiotics was the highest among the knowledge measures, although, in general, the level of knowledge was quite low. The level of awareness and understanding regarding the ways to treat antibiotic resistance was high, but the level of awareness of the severity of antibiotic resistance was medium.

### 2.4. Relationships between Knowledge and Attitudes

The relationships between knowledge and attitudes were analyzed using Pearson correlations. We found positive and significant relationships between the levels of knowledge and attitudes, as shown in Table 4. This indicates that a higher level of knowledge and awareness regarding the severity of antibiotic resistance is associated with a greater awareness and understanding of the means available to treat antibiotic resistance.

### 2.5. Differences between Participant Disciplines

The differences between disciplines in the study variables were analyzed using one-way ANOVA tests. Table 5 highlights the differences between the disciplines with respect to the levels of knowledge and attitudes observed among study respondents. Students in the health sciences expressed the most knowledge of antibiotic resistance and a greater awareness and understanding regarding how to treat antibiotic resistance, followed by students from the social sciences, and computers and management. A follow-up Scheffe test indicated that health science students exhibited significantly more knowledge compared to students in these other disciplines. No differences were found among these disciplines with respect to the level of awareness regarding the severity of antibiotic resistance.

### 2.6. Development of a Linear Regression Model to Predict the Level of Awareness and Understanding Regarding Approaches to Treating Antibiotic Resistance

The results of the multiple linear regression model developed to predict the level of awareness and understanding regarding approaches to treating antibiotic resistance are presented in Table 6. The model included variables that were significantly related to participant attitudes in univariate analyses. The regression was significant (F_(349)_ = 25.43, *p* < 0.001), with an explained variance of 13%. Awareness of the severity of antibiotic resistance is the best predictor of the level of awareness and understanding regarding the ways to treat antibiotic resistance (β = 0.30, *p* < 0.001), followed by general knowledge (β = 0.20, *p* < 0.001).

## 3. Materials and Methods

### 3.1. Research Procedure

This study was a cross-sectional analysis of students enrolled at Ashkelon Academic College in 2023 (about 4000 students). After receiving approval from the Ashkelon Academic College Ethics Committee (approval #40-2023), the questionnaires were programmed using Qualtrics (Qualtrics, Provo, UT, USA) and distributed to the students through email on 12 January 2023. A reminder to complete the questionnaire was sent in the same manner after two weeks. On 11 February 2023, the questionnaire was closed to further participants in the program. The average time taken to answer the questionnaire was 6.1 ± 2.13 min. The introductory page of the questionnaire contained an explanation of the ideas and aims of the questionnaire. Submission of the completed questionnaire represented students’ informed consent to participate in the survey. The students could stop responding to the questionnaire at any stage and had the option to elect not to answer certain questions. No questions were defined as compulsory. There were 524 entries to the questionnaire, of which 371 students filled out at least 90%. Hence, the response rate is 71% of all entries and 9% of the research population. They were divided into 3 groups (disciplines) according to the department they studied in: health sciences, social sciences, and computers and management.

### 3.2. Tools

A professional translator translated the anonymous, closed, self-completed WHO questionnaire, “Antibiotic Resistance: Multi-country public awareness survey” [24] from English into Hebrew. This questionnaire is used among a variety of groups, including student populations, and it has been translated into different languages, e.g., [21,22,24,25,26,27]. After the questionnaire was translated into Hebrew, it was administered to five students who did not attend the college to ensure that the questions were comprehensible. The questionnaire was then revised according to their feedback comments. In addition, two experts in public health and infectious diseases validated the questionnaire using the content validity method. The following describes the questionnaire sections:

1. Demographic information: Gender, age, marital status, religion, department, and year of study.
2. Practice: Three questions: When did you last take antibiotics? Did you get a prescription from a doctor? Did a doctor/nurse/pharmacist explain how to take the antibiotics?
3. Knowledge about the use of antibiotics: Three questions in which respondents were asked to indicate whether, in their opinion, the statement was correct or incorrect or whether they did not know. For example, “You should stop taking antibiotics when you feel well”. The number of correct responses to each statement was totaled to calculate the knowledge score.
4. Knowledge about the necessity of antibiotics for 12 medical conditions: Only 3 of the 12 indicated knowledge of conditions that require antibiotic treatment (gonorrhea, bladder, or urinary tract infection, and skin or wound infection). The number of correct responses to each statement was totaled to calculate the knowledge score.
5. Familiarity with four terms related to antibiotic resistance: Respondents were asked to indicate whether or not they were familiar with the following terms: antibiotic resistance, superbugs, antimicrobial resistance, and antibiotic-resistant bacteria.
6. General knowledge about antibiotic resistance: Eight questions asking respondents to indicate whether, in their opinion, the statement was correct or incorrect or whether they did not know. For example, “Antibiotic resistance is an issue in other countries but not in Israel”.
7. The level of awareness and understanding regarding approaches to treating antibiotic resistance: Seven questions asking respondents to indicate to what extent they agreed with the given statements on a Likert scale ranging from 1 (not at all) to 5 (a very large extent) For example, “People should use antibiotics only when prescribed by a doctor”. The average of the answers was calculated for each participant. Cronbach’s α for reliability was 0.73.
8. Awareness of the severity of antibiotic resistance: Five questions asking respondents to indicate to what extent they agreed with the given statements on a Likert scale ranging from 1 (not at all) to 5 (a very large extent). For example, “Antibiotic resistance is one of the biggest problems the world faces”. The average of the answers was calculated for each participant. Cronbach’s α for reliability was 0.75.

### 3.3. Data Analysis

The data were analyzed using SPSS 29.0 (IBM, Armonk, NY, USA). Relationships between the variables were examined using Pearson correlation analyses. Differences between groups of students were analyzed using χ^2^ tests and one-way analyses of variance (ANOVAs) as appropriate. A linear regression model was used to test the prediction of the level of awareness and understanding regarding the ways to treat antibiotic resistance. All reported *p*-values were based on two-sided tests and were considered significant when the values were below 0.05.

## 4. Discussion

The current study sought to explore knowledge, attitudes, and practices among higher-education students related to antibiotic use and resistance. To the best of our knowledge, this is the first such study conducted among Israeli higher-education students. The overall knowledge level among these students was moderate, and the level of awareness was unsatisfactory, with students from health science disciplines scoring higher than students from social science and computer and management disciplines.

We found that half of the participants reported having used antibiotics in the year prior to the study [22,28]. In contrast, in the United Kingdom, one-third of respondents indicated that they had used antibiotics in the previous year [29]. In line with our findings, some students in the United Kingdom had begun treatment using antibiotics left over from a prior course of treatment without obtaining a new prescription [29]. Although differences among the groups were not significant, this phenomenon was nonetheless more common among students in the health sciences than among students from the other disciplines. It is possible that these health science students are more likely to have family members who work in health professions, thus providing them with better access to drugs without a prescription. Researchers suggest that knowledge among health science students influences their likelihood of taking antibiotics without a prescription [30,31].

We also found that, overall, the level of knowledge and attitudes regarding antibiotic resistance were low. Participant knowledge about the necessity of antibiotic use for the treatment of different medical conditions was rather low (average: 5.75/12), as was the level of knowledge about antibiotic resistance (average: 4.91/8). Comparably, in previous studies, low levels of knowledge about antibiotic resistance were documented among students in different countries [20,22,31,32,33,34]. Moreover, health science students exhibited significantly greater knowledge and were more aware of the problem of antibiotic resistance than social science or computer and management students. When comparing these findings to other studies conducted among university students, our results are consistent with previous surveys [19,20,27,35,36]. With respect to student knowledge of the necessity of antibiotics for the treatment of different medical conditions, the same pattern was observed by Sakr et al. [22], who found that a high percentage of university students agreed that antibiotics could be used to cure colds, fevers, sore throats, and viral infections. These results are consistent with other research highlighting misunderstandings pertaining to antibiotic use [26,37,38]. A lack of understanding of the differences between bacterial and viral infections can contribute to the inappropriate use of antibiotics and to increased antibiotic resistance.

With respect to the familiarity with the four terms related to antibiotic resistance, the average level of knowledge was relatively low (2.52/4). This is consistent with previous research [21,39]. Students from developed countries, including Australia, France, Italy, and other European countries, have also acknowledged their need to learn more about antibiotic resistance [33,37,40,41].

All three groups of students demonstrated a similar level of recognition of the severity of the phenomenon of antibiotic resistance, without significant differences among disciplines, and with all agreeing that antibiotic resistance is a serious problem. These findings are similar to previous studies’ findings [20,28]. It, therefore, seems that despite the differences in the level of knowledge and awareness, all students intuitively understand the severity of the problem of antibiotic resistance.

Moreover, the results of the developed multiple linear regression model indicated that the awareness of the severity of antibiotic resistance is the best predictor of the level of awareness and understanding regarding approaches to treating antibiotic resistance, followed by general knowledge. Previous studies in the field of health behavior have suggested that attitudes are a stronger predictor of behavior than knowledge [42].

### Limitations

The current study has some limitations. First, it was conducted only at Ashkelon Academic College and may not be a representative sample. Second, the groups are not demographically similar, which may cause bias. Third, no causal inferences could be drawn due to its cross-sectional design. Another limitation of the study may be the social desirability bias among the participants.

## 5. Conclusions

These findings provide important insights into the level of understanding of antibiotic resistance among students, which will be helpful when designing interventions aimed at raising knowledge and awareness of antibiotic resistance, its implications, and ways to mitigate it. In order to successfully incorporate lessons related to antibiotic resistance and other public health issues into the higher-education curriculum of non-health disciplines, effective and productive collaboration is required from all the stakeholders: clinicians, universities, governments, drug industries, and the public. All these groups should formulate interventions to address misconceptions regarding how people contribute to antibiotic resistance, and health authorities must improve the oversight of access to antibiotics. A new plan in Israel to fight antimicrobial resistance is currently being considered by the Public Health Services, based on a “one health” approach with a strong emphasis on antimicrobial resistance in food-producing animals [43]. This national plan should also include the component of public education, with an emphasis on students from all disciplines as agents of change.

## Figures and Tables

**Table 1:** Participants’ characteristics.

Characteristics	Sample		Health Sciences		Social Sciences		Computers & Management		χ^2^/F, *p*
	(*n* = 371)		(*n* = 110, 30%)		(*n* = 201, 54%)		(*n* = 60, 16%)		
	*n*	%	*n*	%	*n*	%	*n*	%	
In relationship	210	57	60	55	118	59	32	53	χ^2^ = 2.81, *p* = 0.666
Have children	102	27	21	19	71	35	8	13	χ^2^ = 16.26, *p* < 0.001
Jewish	341	92	90	82	193	96	58	97	χ^2^ = 21.27, *p* < 0.001
Born in Israel	319	86	87	79	178	89	54	90	χ^2^ = 6.24, *p* = 0.044
Year of studies:									
1st 2nd 3rd & 4th	110 201 60	30 54 16	41 85 22	37 42 37	34 74 20	31 37 33	35 42 18	32 21 30	χ^2^ = 5.23, *p* = 0.264
Age (years) (Mean ± SD *)	28 ± 8.58		26 ± 6.22		30 ± 9.41		27 ± 8.55		F = 7.08, *p* < 0.001

* SD = Standard Deviation.

**Table 2:** Antibiotic usage among study participants.

	Sample		Health Sciences		Social Sciences		Computers & Management		
	*(n* = 371)		(*n* = 110, 30%)		(*n* = 201, 54%)		*(n* = 60, 16%)		
	*n*	%	*n*	%	*n*	%	*n*	%	χ^2^, *p*
Last antibiotic use:									
In the last month Last six months Last year More than a year	60 72 55 184	16 19 15 50	22 26 14 48	20 33 13 44	29 35 32 105	14 17 16 52	9 11 9 31	15 18 15 52	χ^2^ = 4.50, *p* = 0.609
Getting a prescription from a doctor:									
Yes Had at home	333 38	90 10	94 16	85 15	185 16	92 8	54 6	90 10	χ^2^ = 3.35, *p* = 0.187
Getting an explanation from a doctor, nurse, or pharmacist	320	86	93	84	175	87	53	87	χ^2^ = 3.08, *p* = 0.544

**Table 3:** Levels of knowledge and attitudes towards antibiotic resistance (*n* = 371).

Variables	Maximum Obtainable Score	Range Obtained by Respondents	Mean ± SD *	Percentage ± SD *
Knowledge about the use of antibiotics	3	0–3	2.19 ± 0.79	73 ± 0.26%
Knowledge about the necessity of antibiotics in medical conditions	12	0–11	5.75 ± 2.75	48 ± 0.23%
Familiarity with four terms related to antibiotic resistance	4	0–4	2.52 ± 1.24	63 ± 0.31%
Knowledge about antibiotic resistance	8	0–8	4.91 ± 2.39	61 ± 0.30%
General knowledge (adding up the scores on all knowledge questions)	27	1–27	15.49 ± 5.35	57 ± 0.20%
The level of awareness and understanding regarding the ways to treat antibiotic resistance	5	2.29–5.00	4.28 ± 0.52	85 ± 0.10%
Awareness of the severity of antibiotic resistance	5	1.67–5.00	3.81 ± 0.68	76 ± 0.13%

* SD = Standard Deviation.

**Table 4:** Relationships between knowledge and attitudes.

Variables	The Level of Awareness and Understanding Regarding the Ways to Treat Antibiotic Resistance	
	*r_p_*	*p*
Knowledge about the use of antibiotics	0.16	0.003
Knowledge about the necessity of antibiotics in medical conditions	0.13	0.015
Familiarity with four concepts related to antibiotic resistance	0.14	0.009
Knowledge about antibiotic resistance	0.14	0.010
General knowledge	0.19	<0.001
Awareness of the severity of antibiotic resistance	0.31	<0.001

**Table 5:** Differences between disciplines.

Variable	Disciplines	*n*	Mean ± SD *	Percentage ± SD *	Range	F	*p*
**General knowledge**	Health Sciences Social Sciences Computers & Management	103 191 56	18.33 ± 4.29 14.54 ± 5.05 13.48 ± 6.11	70 ± 0.16% 56 ± 0.19% 52 ± 0.23%	7–26 2–25 1–25	24.22	<0.001
**Awareness of the severity of antibiotic resistance**	Health Sciences Social Sciences Computers & Management	110 201 60	3.88 ± 0.60 3.76 ± 0.71 3.82 ± 0.73	77 ± 0.12% 75 ± 0.14% 76 ± 0.15%	1.67–5 1.80–5 1.67–5	1.12	0.326
**The level of awareness and understanding regarding the ways of treating antibiotic resistance**	Health Sciences Social Sciences Computers & Management	103 191 56	4.39 ± 0.45 4.25 ± 0.54 4.19 ± 0.57	88 ± 0.09% 85 ± 0.10% 84 ± 0.11%	2.71–5 2.33–5 2.29–5	3.60	0.028

* SD = Standard Deviation.

**Table 6:** Linear regression model for attitudes toward the level of awareness and understanding regarding approaches to treating antibiotic resistance.

Variable	β	B	*p*
General knowledge Awareness of the severity of antibiotic resistance	0.20 0.30	0.02 0.24	<0.001 <0.001
R^2^ Adj. R^2^ * N F_(df)_	0.12 0.13 350 25.43_(349)_		

* Adjusted R-squared: a modified version of R-squared; adds precision and reliability by considering the impact of additional independent variables that tend to skew the results of R-squared measurements.

## Data Availability

The data presented in this study are available on request from the corresponding author.
